# Pressure-induced giant emission enhancement, large band gap narrowing and rich polymorphism in two-dimensional 1,2,4-triazolium lead bromide perovskite†

**DOI:** 10.1039/d4ra07511k

**Published:** 2024-12-05

**Authors:** Mirosław Mączka, Filip Dybała, Artur P. Herman, Waldeci Paraguassu, Antonio José Barros dos Santos, Robert Kudrawiec

**Affiliations:** a W. Trzebiatowski Institute of Low Temperature and Structural Research of the Polish Academy of Sciences Okólna 2 Wroclaw 50-422 Poland m.maczka@intibs.pl; b Department of Semiconductor Materials Engineering, Faculty of Fundamental Problems of Technology, Wrocław University of Science and Technology Wybrzeże Wyspiańskiego 27 Wrocław 50-370 Poland; c Faculdade de Fisica, Universidade Federal do Para Belem 66075-110 Brazil

## Abstract

Layered lead halide perovskites are attractive materials for optoelectronic applications. In this work, temperature-dependent photoluminescence (PL) as well as pressure-dependent Raman and PL studies of lead bromide comprising small disc shape 1,2,4-triazolium cations (Tz^+^) are reported. Temperature-dependent studies reveal that at room-temperature (RT) Tz_2_PbBr_4_ exhibits narrow emission at 2.89 eV related to a free exciton (FE). At low temperature, three new narrow and weakly red-shifted bands as well as a very broad and strongly red-shifted band near 2.2 eV appear. The narrow and broad bands are attributed to self-trapped excitons (STEs) trapped by shallow and deep donors (acceptors), respectively. Pressure-dependent Raman studies revealed the presence of three pressure-induced phase transitions near 2, 6 and 8 GPa to phases II, III and IV, associated with increased distortion of the inorganic subnetwork and apperance of a static disorder in phase IV. These structural changes affect the excitonic emission, which changes from a strong red shift on compression in the ambient pressure I to a weaker red shift in phase II, negligible shift in phase III and blue shift in phase IV. Moreover, a new narrow and weakly red-shifted band appears in phases II–IV. Most importantly, PL intensity increases 16.7 times when pressure changes from ambient to 7.77 GPa but decreases in phase IV. The increase in PL intensity can be attributed to the increase in STE formation energy and activation energy for non-radiative recombination, while the decrease in intensity may be related to the formation of point defects, which are the source of non-radiative recombination. Overall, high-pressure PL data show that application of external pressure allows band gap engineering and giant enhancement of the PL efficiency of Tz_2_PbBr_4_ perovskite.

## Abstract 2

Application of external pressure leads to three pressure-induced phase transitions, allows band gap engineering and induces giant enhancement of the photoluminescence efficiency of the Tz_2_PbBr_4_ perovskite.

## Graphical abstract

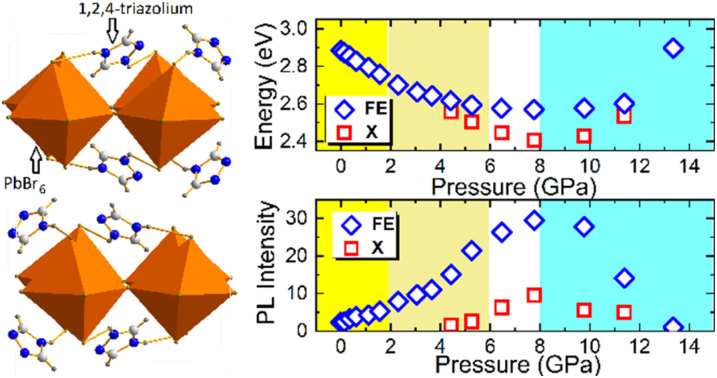

## 1. Introduction

Hybrid organic–inorganic lead halides received enormous interest in recent years as promising materials for a plethora of applications, including solar cells, light-emitting and ferroelectric devices, dielectric and nonlinear-optical (NLO) switches, photodetectors and many more.^1–12^ The wide range of functional properties exhibited by these compounds stems from their remarkable structural versatility. In particular, these compounds may crystallize in different dimensionalities (three-dimensional (3D), two-dimensional (2D), one-dimensional (1D) or even zero-dimensional (0D)) and present different octahedral connectivity (corner-, edge- or face-sharing).^1–13^ Among these, 2D layered analogues constitute the largest subclass of hybrid lead halides. The most common analogues of general formula A_2_PbX_4_ and A′PbX_4_, where A and A′ denote monovalent and divalent organic cations, respectively, and X stands for halogen anion, are composed of inorganic perovskite layers separated by organic cations.^2,3,5,8^ The main advantage of 2D analogues is their improved chemical stability compared to their 3D counterparts.^2,3,5,8^ The dielectric confinement induced by presence of the organic layers leads, however, to significant widening of the band gap and increase of the exciton binding energy.^2,3,5,8^ These features make 2D halides less useful for photovoltaic applications but are beneficial for PL performance. Furthermore, contrary to narrow PL arising from radiative recombination of free excitons (FE) exhibited by 3D analogues, 2D counterparts may also show broadband emission usually attributed self-trapped excitons (STEs).^5–10,13–15^ In some instances, this broadband emission may be white, making 2D perovskites attractive for lighting.^16,17^ Furthermore, 2D analogues may also display other functional properties induced by loss of an inversion centre such as ferroelectricity,^7,8^ giant piezoelectricity,^18^ or second-harmonic generation (SHG).^8,19^

It is well-known that temperature-induced structural changes (*e.g.*, Pb–X bond lengths and distortion of the inorganic framework) and dynamics of organic cations significantly affect optoelectronic properties of hybrid lead halides.^3,4,20^ Moreover, some functionalities like NLO-switching or ferroelectricity rely on presence of temperature-induced structural phase transitions associated with loss of an inversion centre and appearance polar phases (for ferroelectricity).^4,7–10^ External pressure is another thermodynamic parameter that can be used for modifying structural and optoelectronic properties.^21–31^ It is important to note that the effect of pressure on structural parameters is much stronger compared to temperature, *i.e.*, hydrostatic pressure can cause a modification of the crystal structure (*i.e.*, bond lengths and angles) much greater than that resulting from temperature changes, and moreover, this is not accompanied by a change in the thermal energy in the system. On the other hand, the emission efficiency strongly depends on temperature, because thermal energy can activate nonradiative recombination processes, which are associated with the presence of point defects or other crystal imperfections. Therefore, compression cause profound implications in the optoelectronic properties of these materials. Furthermore, it can lead to appearance of new polymorphs not available under ambient pressure, which may exhibit enhanced or new functional properties.^21,25,26,28–30^ In addition, it is possible that high hydrostatic pressure may promote the generation of point defects or other crystal imperfections that are responsible for optical degradation and weaken the quantum efficiency of emission. Therefore, when studying the effect of pressure on optical properties, it is good to know the effect of temperature on these properties as well.

The short discussion presented above shows that in order to understand structural and optoelectronic properties of hybrid lead halides, it is important to perform both temperature- and pressure-dependent studies. Temperature-dependent studies are very common but high-pressure studies of 2D lead halide perovskites are still relatively sparse. Nevertheless, the high-pressure studies of several (001)-derived A_2_PbX_4_ analogues with large organic cations, such as PEA_2_PbBr_4_, PEA_2_PbI_4_, BA_2_PbBr_4_ and PA_2_PbBr_4_ (PEA = phenethylammonium, BA = butylammonium, PA = propylammonium) revealed very similar behaviour for these compounds, *i.e.*, band gap narrowing on compression up to 5–12 GPa followed by band gap widening on further compression.^21–24^ The narrowing of band gap was attributed to dominating effect of Pb–X bonds shortening at lower pressure regime and severe octahedral distortion at higher pressure regime, related to pressure-induced phase transitions to strongly distorted phases.^21–24^ Interestingly, the strong distortion of the inorganic subnetwork lead in some instances to appearance of STEs-related broadband emission and very large enhancement of PL.^25–27^ The number of lead halide perovskites exhibiting large PL enhancement on compression is, however, still very small. High-pressure studies of 2D perovskites comprising small cations are very rare and to our best knowledge they were reported only for only two (001)-oriented perovskites (MHy_2_PbX_4_ (X = Br, I)), GA_2_PbI_4_, which comprises double layers of corner-sharing octahedra, and corrugated (110)-oriented IMMHyPbBr_4_ (GA^+^ = guanidinium, MHy^+^ = methylhydrazinium and IM^+^ = imidazolium).^28–31^ These studies also revealed band gap narrowing at lower pressure regime followed by band gap widening at high pressures (the only exception is IMMHyPbBr_4_, which showed continuous but less pronounced narrowing also in the high-pressure phase up to 7.2 GPa).^28–31^ However, we showed that for small cations a new type of pressure-induced phase transition may be realized. This behaviour was observed for MHy_2_PbBr_4_ and MHy_2_PbI_4_, which undergo near 4 and 3 GPa, respectively, the structural phase transition associated with extrusion of organic cation from the interlayer space to the intralayer perovskite voids.^29,30^ Interestingly, this unusual transformation resulted in giant increase of PL intensity (57-fold for the bromide) and unprecedented negative compressibility within the layers.^29,30^

One subgroup of lead halides, which attracted attention in recent years due their structural and optical properties as well as birefringence switching, are those comprising small disc-shaped cations such as IM^+^, acetamidinium (ACE^+^) or 1,2,4-triazolium (Tz^+^).^32–36^ Literature data showed that IM_2_PbBr_4_ adopts a 1D double chain structure and exhibit broadband bluish-green emission.^36^ For the remaining cations, 2D structures are formed: a (110)-oriented structure for ACE_2_PbBr_4_ and (001)-oriented structure for Tz_2_PbX_4_ (X = Cl, Br).^32–35^ Tz_2_PbCl_4_ showed both narrow FE-related and broadband STEs-related emissions, and pronounced thermochromism.^35^ Interestingly, Tz_2_PbBr_4_ can be obtained at RT in two different polymorphs, *C*2/*c* or *P*2_1_/*c*, which have similar a near-eclipsed neighbouring layers but the former polymorph comprises Tz^+^ cations ordered over two distinct sites whereas the latter presents a single Tz^+^ site.^32,33^

Herein, we report temperature- and pressure-dependent PL data for the *C*2/*c* polymorph of Tz_2_PbBr_4_. We also performed pressure-dependent Raman studies, which provided information on pressure-induced structural changes and mechanism of the three discovered phase transitions. The structural information obtained from Raman studies also allowed to better understand origin of pressure-induced changes in PL properties.

## 2. Experimental section

### 2.1 Synthesis

Tz_2_PbBr_4_ crystals were grown by evaporation method, reported previously by Gu *et al.*^32^ The reaction mixture was prepared by dissolving 2 mmol of PbBr_2_ (98%, Sigma-Aldrich) and 5 mmol of 1,2,4-triazole (98%, Sigma-Aldrich) in HBr under stirring on a hot plate (60 °C). Then heating was switched off and the cooled solution was left at RT for slow evaporation. Transparent pale yellow crystals were separated from the mother liquid after 3 days and dried at RT. Their powder X-ray diffraction pattern is in good agreement with the calculated one based on the RT *C*2/*c* structure of polymorph I reported by Guo *et al.*^32^ (Fig. S1, ESI†), confirming purity of the bulk sample.

### 2.2 Powder X-ray diffraction (PXRD)

PXRD patterns of the ground crystals were measured in the reflection mode using an X'Pert PRO X-ray diffraction system equipped with a PIXcel ultrafast line detector and Soller slits for CuKα_1_ radiation (*λ* = 1.54056 Å).

### 2.3 Thermogravimetric (TG) measurement

Thermogravimetric (TG) study was performed in the temperature range 300–1200 K using a PerkinElmer TGA 4000. The sample weight was 78.03 mg and the heating speed rate was 10 K min^−1^. Pure nitrogen gas as an atmosphere was used.

### 2.4 Optical absorption and PL studies

RT diffuse reflectance spectrum of the powdered sample was measured using the Varian Cary 5E UV-vis-NIR spectrophotometer. For PL measurements with temperature, the sample was placed in a closed-cycle cryostat operating in 20–320 K temperature range. The sample was excited by a 325 nm line from HeCd laser (Kimmon IK3501R-G) with the power density of 0.2 W cm^−2^. PL signal was collected with Peltier-cooled Avantes Si CCD spectrometer. For PL measurements under hydrostatic pressure, the sample was mounted in diamond anvil cell (DAC, application of DACs in physical investigations can be found in ref. 37 and 38). Diacell® CryoDAC-nitro from Almax-EasyLab was used.^39^ The pressure in this cell was determined using the shifts in the R1 and R2 fluorescence lines of ruby spheres.^40,41^ A 325 nm HeCd laser was used to excite the sample and ruby spheres. The excitation density was about 1 W cm^−2^ before DAC. The PL signal was collected from DAC with the appropriate optics and analyzed using a 0.5 m Andor monochromator with a 150 g mm^−1^ diffraction grating blazed at 500 nm and a Si CCD camera cooled down to −70 °C by Peltier elements.

### 2.5 High-pressure Raman spectroscopy

High-pressure Raman spectra were acquired using a LabRAM spectrometer (HR evolution from Horiba) equipped with a thermoelectrically cooled charge-coupled device (CCD) detection system and a diamond anvil cell (DAC) μ-scope HT(S)^42^ from Almax-EasyLab. The sample was loaded into a 100 μm diameter hole, spark-eroded in a 200 μm thick stainless steel gasket using driller from Almax-EasyLab.^43^ Pressure was determined *via* the shifts in the R1 and R2 fluorescence lines of ruby.^40,41^ Mineral oil (Nujol) was employed as the pressure-transmitting medium. A red He–Ne laser (*λ* = 633 nm) was used as the excitation source in a backscattering geometry, with a laser power of 3.0 mW incident on the sample. Each spectrum was recorded over 70 seconds with three accumulations, and a spectral resolution of 2 cm^−1^.

## 3. Results and discussion

### 3.1 TG

The TG plot shows that Tz_2_PbBr_4_ starts to decompose near 520 K (Fig. S2†). The first weight loss of ∼43.7%, which finishes near 650 K, can be attributed to the release of 1,2,4-triazolium bromide (the calculated value is 45.0%). The second weight loss, which starts near 800 K and finishes near 1000 K, is due to sublimation of PbBr_2_.

### 3.2 Raman scattering studies

**Fig. 1:**
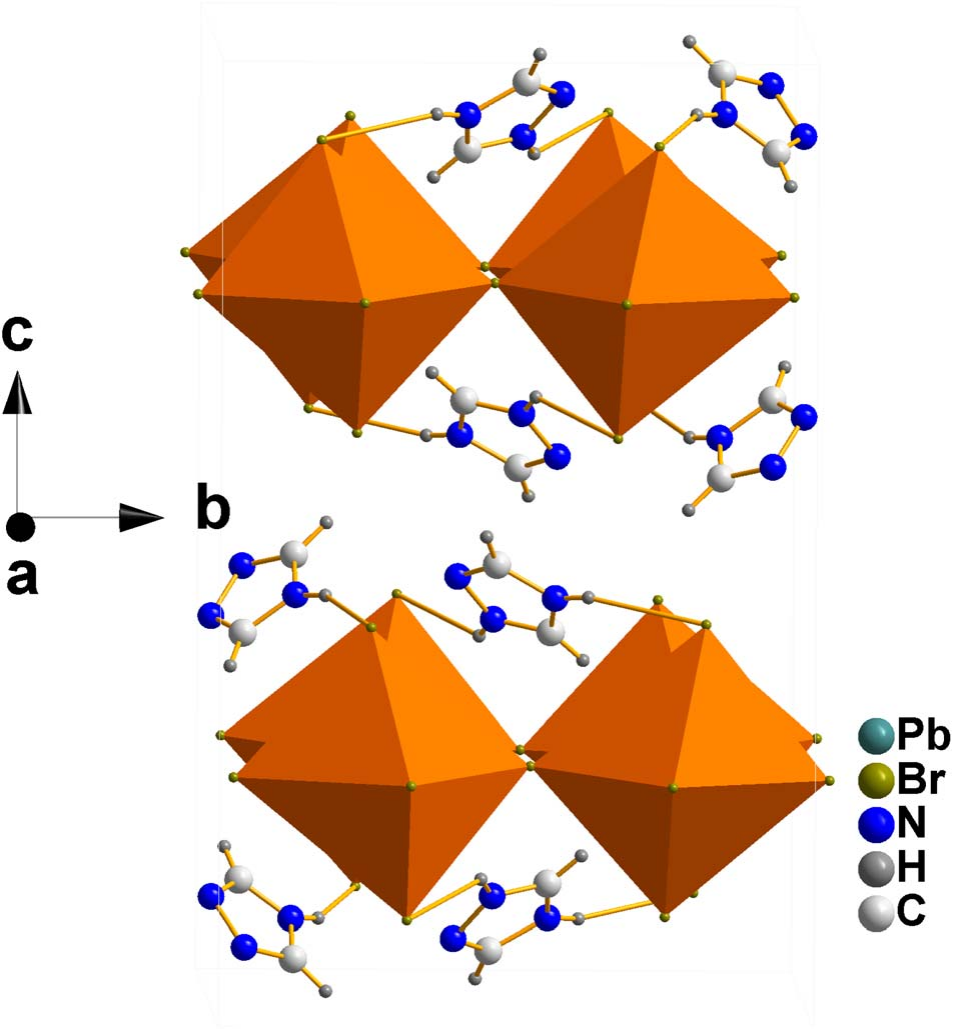
Crystal structure of Tz_2_PbBr_4_ at 298 K (polymorph I, *C*2/*c* structure) along the *a*-direction showing octahedral layers and ordered IM^+^ cations located at the interlayer space.

Before we proceed to discussion of Raman data, it is important to present shortly crystal structure of Tz_2_PbBr_4_. This compound crystallizes at RT in the monoclinic *C*2/*c* phase (Fig. 1). It comprises single-layer octahedral sheets built up of corner-shared PbBr_6_ units. The ordered Tz^+^ cations separate the inorganic layers and the unit cell comprises two unique cations.^32^ The PbBr_6_ octahedra are tilted leading to significant departure of the Pb–Br–Pb angles from 180° (to 162.94(4), 165.82(3) and 169.44(4)° at 298 K).^32^

**Fig. 2:**
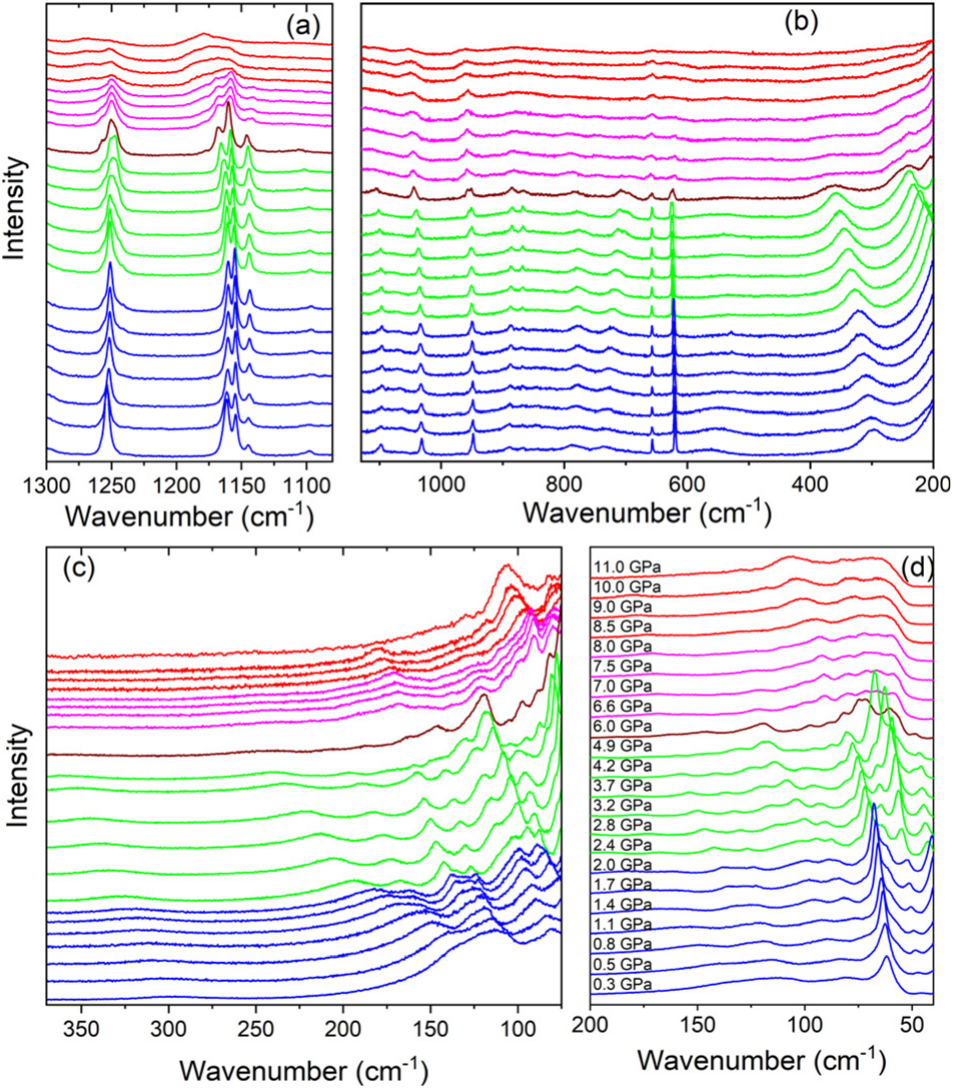
Raman spectra of Tz_2_PbBr_4_ recorded during compression in the (a) 1300–80 cm^−1^, (b) 1130–200 cm^−1^, (c) 370–75 cm^−1^ and (d) 200–40 cm^−1^ ranges. Blue, green, magenta and red colours correspond to the ambient pressure phase I and the HP phases II, III and IV, respectively. The spectrum at 6.0 GPa was denoted in wine colour since it corresponds to mixed II and III phases.

Raman spectra of Tz_2_PbBr_4_ on compression and pressure dependence of Raman wavenumbers are presented in Fig. 2 and S3,† respectively. The values of wavenumber intercepts at zero pressure (*ω*_0_) and pressure coefficients (*α* = d*ω*/d*P*) obtained from fitting of the experimental data with a linear function *ω*(*P*) = *ω*_0_ + *αP* are listed in Table S1.† The assignment of internal modes of Tz^+^ cation presented in Table S1† is based on experimental and theoretical studies of Tz_2_PbCl_4_ and bis(1,4-H_2_-1,2,4-triazolium) hexachloridostannate(iv) monohydrate.^35,44^ The assignment of lattice modes is proposed based on experimental data for a number of (001)-oriented layered lead bromide perovskites.^45,46^

According to the crystallographic data, the ambient pressure *C*2/*c* phase I comprises Tz^+^ cations ordered over two distinct sites.^32^ In spite of this feature, splitting into doublet is observed clearly only for the *γ*_ring_ CNCN mode (620.3 + 618.8 cm^−1^ doublet, Fig. S3 and Table S1†). This behaviour points to very similar structures of the both unique Tz^+^ cations. Raman spectra do not show any signs of a structural phase transition up to 2.0 GPa but some continuous changes in intensity of the lattice modes can be clearly seen (Fig. 2c and d). Table S1† shows strongly negative pressure coefficients α for the γN–H modes observed near 736 and 782 cm^−1^. This behaviour indicates large increase of the hydrogen bond strength on compression. The remaining internal modes exhibit weak shifts on compression but very large (up to 23.33 cm^−1^ GPa^−1^) and positive values of pressure coefficients α are observed for the lattice modes (Table S1†). This behaviour proves that pressure weakly affects Tz^+^ structure but has strong effect on Pb–Br bond lengths and Pb–Br–Pb angles. Thus, our results indicate that external pressure is accommodated mainly through decrease of the interlayer distance, which leads to increase of the hydrogen bond strength between Tz^+^ and the bromine anions, causing increased tilting of the PbBr_6_ units. We have observed similar behaviour for (001)-oriented MHy_2_PbBr_4_ and (110)-oriented IMMHyPbBr_4_.^29,31^ However, whereas the largest coefficients α of the former compound are only slightly smaller compared to Tz_2_PbBr_4_,^29^ they are about two times smaller for IMMHyPbBr_4_.^31^ This behaviour proves that the mechanical softness of (001)-oriented perovskites is higher compared to (110) analogues.

When pressure increases beyond 2 GPa, the Raman spectra exhibit significant changes, which can be attributed to a pressure-induced phase transition into a high-pressure phase II. First of all, the δC–H, δNCN and γN–H modes observed as singlets near 1251.0, 949.3 and 722.3 cm^−1^ at 2.0 GPa split into doublets above 2.4 GPa (Fig. 2a, b and S3, Table S1†). Since the ambient pressure phase I contains two unique Tz^+^ cations, the observed behaviour suggests that not resolved bands in phase I becomes resolved in phase II due to increased difference in the internal structure of the both Tz^+^ cations. Another indication of the phase transition is changes of the pressure dependence of some internal modes from negative in phase I to positive in phase II (Table S1†). However, relative intensities and wavenumbers of the Raman bands of Tz^+^ cations do not show any significant changes at the phase transition. Raman data indicate, therefore, that this phase transition weakly affects bond length of the Tz^+^ cations. Contrary to weak pressure-induced changes of the internal modes, the phase transition leads to pronounced changes of the lattice modes (Fig. 2c and d). Firstly, lattice modes show very strong changes of the relative intensities (see for instance the bands near 52 and 65 cm^−1^, Fig. 2d). Furthermore, some lattice bands exhibit sudden shifts at the phase transition (see Fig. S3†). These features indicate that this phase transition is associated with pronounced tilts and/or distortion of the PbBr_6_ octahedra. Secondly, majority of lattice bands, especially those in the 170–90 cm^−1^ range, exhibit very pronounced narrowing (Fig. 2c and d). Such behaviour points to strong decrease of the lattice dynamics in phase II. Thirdly, nearly all lattice modes exhibit significant decrease of the pressure coefficients α when going from phase I to phase II (Table S1†), indicating weaker compressibility of the latter phase.

On further increase of pressure, significant changes in the Raman spectra become obvious at 6.0 GPa but closer inspection indicates that at this pressure, two high-pressure phases II and III coexists, and the transformation to phase III is finished at 6.6 GPa. This behaviour points to strongly first-order character of this transformation due to strong reorganization of the crystal structure. Indeed, Raman spectra in the lattice modes region show that when phase II changes to phase III, the spectra exhibit spectacular changes in relative intensities of Raman bands (see Fig. 2c and d). These changes are associated with shifts, observed also for the internal modes (Fig. 2 and S3†), and a few-fold decrease in the values of the pressure coefficients (Table S1†). The number of Raman bands remains approximately the same in phases II and III. Overall changes in the Raman spectra indicate that the transformation to phase III is associated with further distortion of the PbBr_6_ octahedra and reorientation of Tz^+^ cations, which result in much denser and compact crystal structure.

Raman bands in the lattice modes region exhibit weak changes up to 8.0 GPa but when pressure increases above 8.5 GPa, the bands exhibit strong broadening (Fig. 2c and d). We suppose, therefore, that Tz_2_PbBr_4_ exhibits the third pressure-induced phase transition between 8.0 and 8.5 GPa. The broadening is also observed in the internal modes region (Fig. 2a and b). This behaviour suggests that phase IV is disordered but since positions and relative intensities of Raman bands remain similar at 8.0 and 8.5 GPa, the crystal structures of phases III and IV are similar. It is worth noting that significant broadening of Raman and/or diffraction peaks at very high pressures was previously reported for a number of lead halide perovskites and this type of static disorder was attributed to distortion of both organic cations and octahedral units.^47–49^ The pressure at which this disorder is observed for Tz_2_PbBr_4_ is very similar to that reported for (C_6_H_5_CH_2_CH_2_NH_3_)_2_PbBr_4_ (8.2 GPa).^47^

Fig. S4† shows that all phases reappear during decompression experiment. This behaviour proves that the pressure-induced phase transitions are reversible.

### 3.3 Temperature-dependent optical studies

**Fig. 3:**
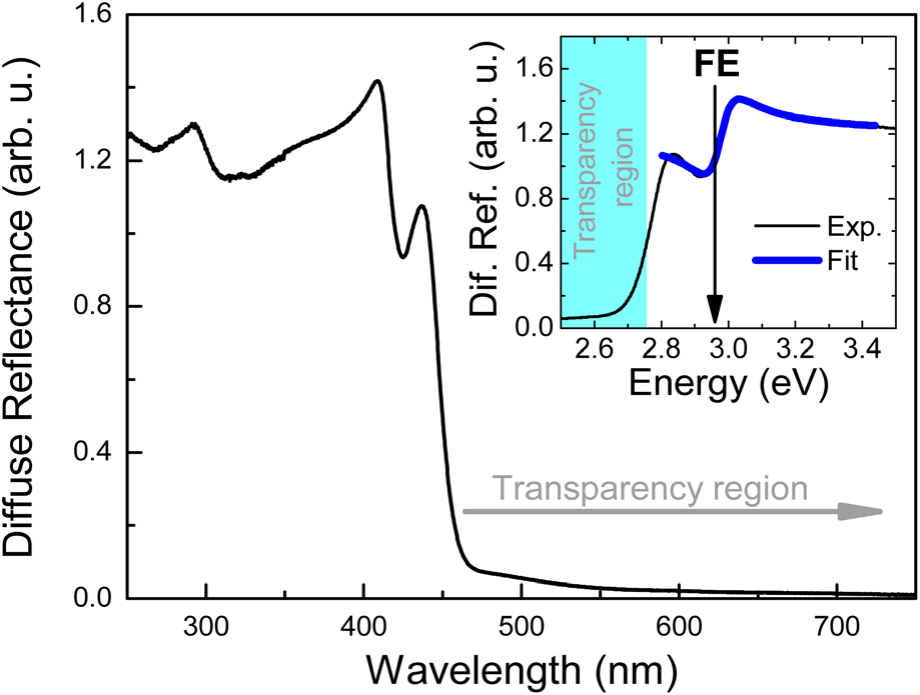
The diffuse reflectance spectrum of Tz_2_PbBr_4_. The inset shows an excitonic transition fitted by a theoretical formula for an excitonic resonance in reflectance.

Fig. 3 shows RT diffuse reflectance (DF) spectrum of Tz_2_PbBr_4_. Above ∼450 nm (*i.e.*, in visible range) this crystal is transparent and therefore DF achieves very low value. Below 450 nm an excitonic transition is clearly visible. This transition is plotted on an energy scale (see inset) and fitted using the well-known semiconductor formula for the excitonic resonance in the reflectance given by eqn (1)1
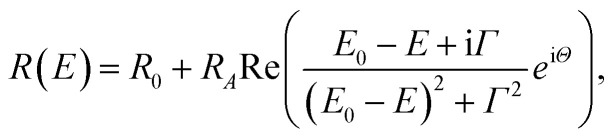
where *R*_A_, *E*_0_, and *Γ* is an amplitude, energy, and broadening of FE transition. *R*_0_ is a background and *Θ* is a phase of this transition. The energy of excitonic transition extracted from this fit is 2.96 ± 0.02 eV and its broadening is 50 ± 10 meV. The band gap of this perovskite should be higher by 100–200 meV as the exciton binding energy from this range is expected for 2D perovskites. This means that band gap value is comparable to the band gaps of other (001)-oriented lead bromide perovskites, typically observed near 3–3.2 eV.^5,8,50^

**Fig. 4:**
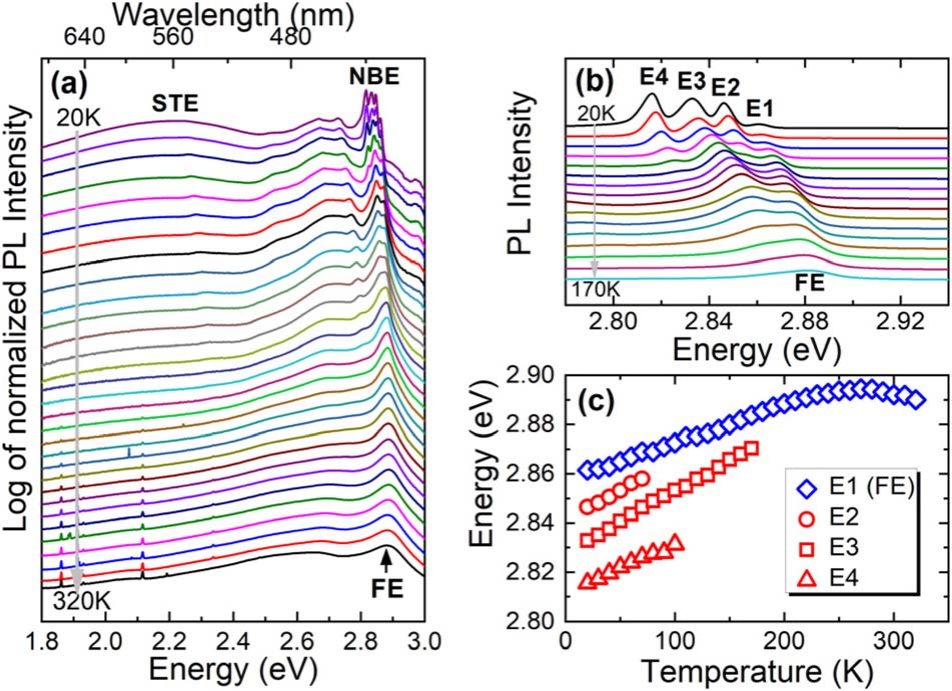
(a) PL spectra measured over a wide spectral range at various temperatures (for better visualization of weak bands, the logarithm of normalized PL was plotted). (b) PL spectra measured in the NBE region at various temperature. (c) Spectral positions of E1 (FE), E2, E3, and E4 peak with temperature.

Fig. 4a and b show the temperature dependence of the PL spectra measured over a wide spectral range and the enlargement of the near-bandgap emission (NBE), respectively. The NBE emission consists of four bands at very low temperatures, which are numbered E1, E2, E3, and E4 going from higher to lower energy. The spectral position of these bands systematically shifts towards higher energies with increasing temperature, see Fig. 4c. This is related to the opening of the energy gap with increasing temperature, which is typical for many perovskites.^51–54^ The intensity of the E2, E3 and E4 bands decreases very quickly with increasing temperature and therefore these peaks can be attributed to excitons bound on shallow donors (acceptors). But in 2D perovskites we are dealing with STEs because of strong local lattice distortions around the photoexcitation.^15,16,55^ Therefore, we attribute bands E2, E3, and E3 to STEs which are additionally trapped by shallow donors (acceptors). STEs can also be created near a deep donor (acceptor) and thereby their energy can be much lower than the band gap.^16^ The wide emission band with a maximum at 2.2 eV is attributed to such emission. The self-trapping mechanism weakens as the thermal energy increases, and therefore a FE emission is observed at RT and dominates at this temperature (E1 band at low temperatures).

To summarize the PL measurements with temperature, the evolution of the PL spectra does not indicate a phase transition in this temperature range, and the presence of the E2–E4 peaks indicates the presence of donor and/or acceptor states in the studied crystals, which can be sources of non-radiative recombination at room temperature. These states may be caused by unintentional impurities and/or natural point defects, the nature of which is difficult to determine unambiguously. The increase in temperature causes an increase in thermal energy in the studied crystals and the activation of non-radiative recombination processes, which causes a decrease in PL intensity (the intensity can be traced in the PL spectra in Fig. 4b) and is commonly observed not only for perovskites. In the case of perovskites, it is worth emphasizing that we are dealing here with the process of exciton self-trapping induced by the deformation of the lattice after the generation of an electron–hole pair. The decrease in STE intensity may indicate that the exciton self-trapping process weakens with increasing temperature, and this may be caused by (i) an increase in thermal energy and (ii) a change in polaron formation energy, which is responsible for the exciton self-localization process. PL measurements at constant temperature (constant thermal energy) and variable hydrostatic pressure allow us to look at PL intensity from a perspective in which thermal energy does not change. The change in PL intensity in this case will be due to the change in exciton self-trapping energy (higher energy – higher intensity) and the change in defect concentration, which are the source of non-radiative recombination (increase in defect concentration – decrease in PL intensity) caused by hydrostatic pressure.

### 3.4 Pressure-dependent optical studies

**Fig. 5:**
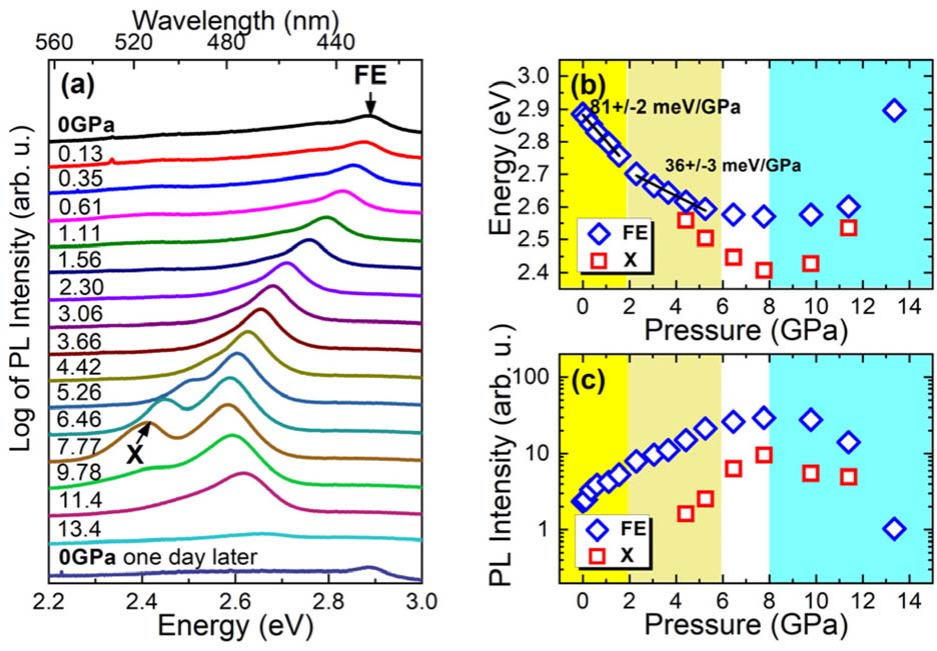
(a) Photoluminescence spectra of Tz_2_PbBr_4_ measured under different hydrostatic pressure at room temperature (for better visualization log of photoluminescence is plotted). Spectral position of FE and X peaks (b) and their intensities (c).

Fig. 5a shows PL spectra of Tz_2_PbBr_4_ measured under different hydrostatic pressure at RT. As the pressure increases, the FE emission shifts to the red. This shift is linear up to a pressure of ∼2 GPa with a ratio of 81 ± 2 meV GPa^−1^ (see Fig. 5b) and is accompanied by an increase in intensity for this emission, see Fig. 5c. It is well-known that the shortening of Pb–X bonds leads to red shift of the excitonic absorption and thus FE emission due to increased antibonding interaction in the conduction band maximum whereas increased octahedral tilting has an opposite effect due to weakening of the interaction between Pb s orbitals and halide p_*x*_ and p_*y*_ orbitals in the valence band maximum as well as the mixing of halide p and s orbitals.^29,56–58^ The observed red shift of the FE emission band indicates, therefore, that up to ∼2 GPa pressure leads to significant shortening of Pb–Br bonds but weakly affects PbBr_6_ tilting. The red shift continues in the 2–6 GPa range but with a smaller ratio of 36 ± 3 meV GPa^−1^ (see Fig. 5b). This behaviour suggests that in the 2–6 GPa range pressure induces significant tilts and distortion of the octahedral units. The assumption is consistent with the Raman data, which revealed presence of a pressure-induced phase transition into a high-pressure phase II near 2 GPa, associated with distortion of the inorganic sublattice. It is worth noting that at 4.42 GPa a new PL band X becomes visible, which at this pressure is red shifted compared to the FE one by about 58 meV (Fig. 5a–c). This new band can be most likely attributed to STEs induced by hydrostatic pressure. This may mean that the polaron formation energy in this crystal increases with increasing hydrostatic pressure. Stronger self-trapping of excitons favors the increase of PL intensity because such excitons are less mobile than FE and therefore will be less trapped and dissociated by nonradiative recombination centers. In our measurements, this emission (X peak in Fig. 5a) is clearly visible above the pressure of ∼4 GPa, but its participation at lower pressures is not excluded. Moreover, the pressure-induced spectral shift of FE emission can increase the activation energies for non-radiative processes and hence the PL intensity can also increase. It is worth noting here that an increase in hydrostatic pressure, similarly to a decrease in temperature, causes a red shift of FE emission due to the change in bond lengths and angles and these processes are accompanied by an increase in PL intensity.

In the 6–8 GPa pressure range, a further increase in emission intensity is observed but the FE band does not show any significant shift. This behaviour indicates that the effect of Pb–Br bond shortening on compression is compensated by the effect of PbBr_6_ tilting and distortion. This behaviour is in good agreement with presence of the second pressure-induced phase transition near 6 GPa to a more distorted and much denser phase III. It is worth noting that intensity of both FE and X bands reaches maximum at 7.77 GPa. When pressure increases from ambient to 7.77 GPa, intensity of the FE band increases 12.6 times and the total intensity of the both bands shows increase of 16.7 times. The intensity enhancement is smaller than reported for (001)-oriented MHy_2_PbBr_4_ (57-fold)^29^ but larger than for majority of other layered lead halide perovskites, for instance (GA)(MA)_2_Pb_2_I_7_ (5-fold, MA^+^ = methylammonium)^59^ or (3AMP)PbI_4_ (about 10-fold, 3AMP^2+^ = -3-aminomethyl pyridinium).^60^ Our results show, therefore, that emission properties of Tz_2_PbBr_4_ improve on the applied external pressure in a very broad 0–7.77 GPa range.

From a pressure of ∼8 GPa, pressure dependence of the FE band changes to a blue shift. In addition, a decrease in emission intensity is observed above 8 GPa. The X band, which appeared as a result of the applied pressure, see Fig. 5a, behaves very similarly. Hence we can conclude that around 8 GPa we are dealing with a third phase transition to another phase. The blue shift indicates that in this new phase pressure-induced octahedral tilting and distortion prevails over pressure-induced bond shortening. Thus the emission data corroborate with Raman results, which revealed pressure-induced phase transition between 8 and 8.5 GPa to a new phase with significant static disorder. This disorder seems to be responsible for the observed decrease of the emission intensity (Fig. 5b and c) and broadening of the FE and X bands (Fig. 5a) above 8 GPa.

In general, we expect that the increase in hydrostatic pressure can induce degradation of perovskites through the formation of natural point defects, which should manifest itself in a decrease in PL intensity, and the probability of such a process should be higher with increasing pressure. This phenomenon cannot be completely ruled out for the studied crystals, but the increase in PL intensity with increasing pressure indicates that such a process is not significant up to at least ∼6–8 GPa, and the increase in PL intensity may be due to earlier mentioned processes (*i.e.*, pressure-induced increase in polaron formation energy (exciton self-trapping energy) and activation energy for non-radiative processes).

Some increase in PL intensity associated with the weakening of non-radiative recombination processes in a system with constant thermal energy (*i.e.*, measurements at variable hydrostatic pressure and constant temperature) can also be obtained by reducing the concentration of non-radiative recombination centres, but such a phenomenon is not expected in this case.

## 4. Conclusions

Temperature-dependent PL studies of Tz_2_PbBr_4_ showed that RT PL is dominated by that arising from FE recombination. This emission exhibits red shift with decreasing temperature indicating that on cooling the shortening of the Pb–Br bonds prevails over octahedral tilting. We also show that at low temperatures three new, narrow and weakly red-shifted components appear that can be attributed to STEs trapped on shallow donors (acceptors). Tz_2_PbBr_4_ shows also presence of a very broad and strongly red-shifted band near 2.2 eV at low temperatures that arises from STEs trapped on deep donors (acceptors).

Pressure-dependent Raman studies reveal that Tz_2_PbBr_4_ is strongly compressible in the low pressure regime due to weak interactions in the direction perpendicular to the layers. The increased hydrogen-bond strength on compression leads to a first phase transition near 2 GPa, associated with tilts and distortion of the PbBr_6_ octahedra. The second phase transition is revealed near 6 GPa through large modification of the Raman spectra. Raman data provide evidence that this structural modification leads to much denser and compact phase with significantly distorted PbBr_6_ octahedra. As a result, the FE band shows negligible pressure dependence in the 6–8 GPa range. The final pressure-induced phase transition occurs near 8 GPa into significantly disordered phase, as evidenced by broadening of Raman and PL bands as well as blue shift of the FE band. Overall, our results show that Tz_2_PbBr_4_ exhibits three pressure-induced phase transitions and giant increase of PL intensity on compression up to 7.77 GPa. Thus, application of external pressure is an effective way for band gap engineering and giant improvement of the PL efficiency of this perovskite.

## Data availability

The data supporting this article have been included as part of the ESI.†

## Author contributions

Conceptualization: M. M. Data curation: W. P., A. J. B. S., F. D. and A. P. H. Formal analysis: M. M., W. P., A. J. B. S., F. D., and A. P. H. Investigation: W. P., A. J. B. S., F. D. and A. P. H. Methodology: all authors. Resources: M. M. Supervision: M. M. and R. K. Validation: M. M. and R. K. Writing – original draft: all authors. Writing – review and editing: all authors. All the authors have given their approval to the final version of the manuscript.

## Conflicts of interest

There are no conflicts to declare.

## Supplementary Material

RA-014-D4RA07511K-s001

RA-014-D4RA07511K-s002
