# Supplementary material for: Pressure-induced giant emission enhancement, large band gap narrowing and rich polymorphism in two-dimensional 1,2,4-triazolium lead bromide perovskite
Source: RSC Adv. 2024 Dec 5;14(52):38514–22. doi: 10.1039/d4ra07511k (PMC11618524; doi:10.1039/d4ra07511k)
Supplement: RA-014-D4RA07511K-s002 [file RA-014-D4RA07511K-s002.pdf]

Supplementary Information (SI) for RSC Advances.
This journal is © The Royal Society of Chemistry 2024

Supporting information for

Pressure-induced giant emission enhancement, large band gap narrowing and rich polymorphism in two-dimensional 1,2,4-triazolium lead bromide perovskite

Mirosław Mączka,*[a] Filip Dybała,[b] Artur P. Herman,[b] Waldeci Paraguassu,[c] Antonio José Barros dos Santos,[c] Robert Kudrawiec[b]

[a]*W. Trzebiatowski Institute of Low Temperature and Structure Research, Polish Academy of Sciences, ul. Okólna 2, 50-422 Wrocław, Poland*

[b]*Department of Semiconductor Materials Engineering, Faculty of Fundamental Problems of Technology, Wrocław University of Science and Technology, Wybrzeże Wyspiańskiego 27, 50-370 Wrocław, Poland*

[c]*Faculdade de Fisica, Universidade Federal do Para, 66075-110 Belem, Brazil*

e-mail: m.maczka@intibs.pl

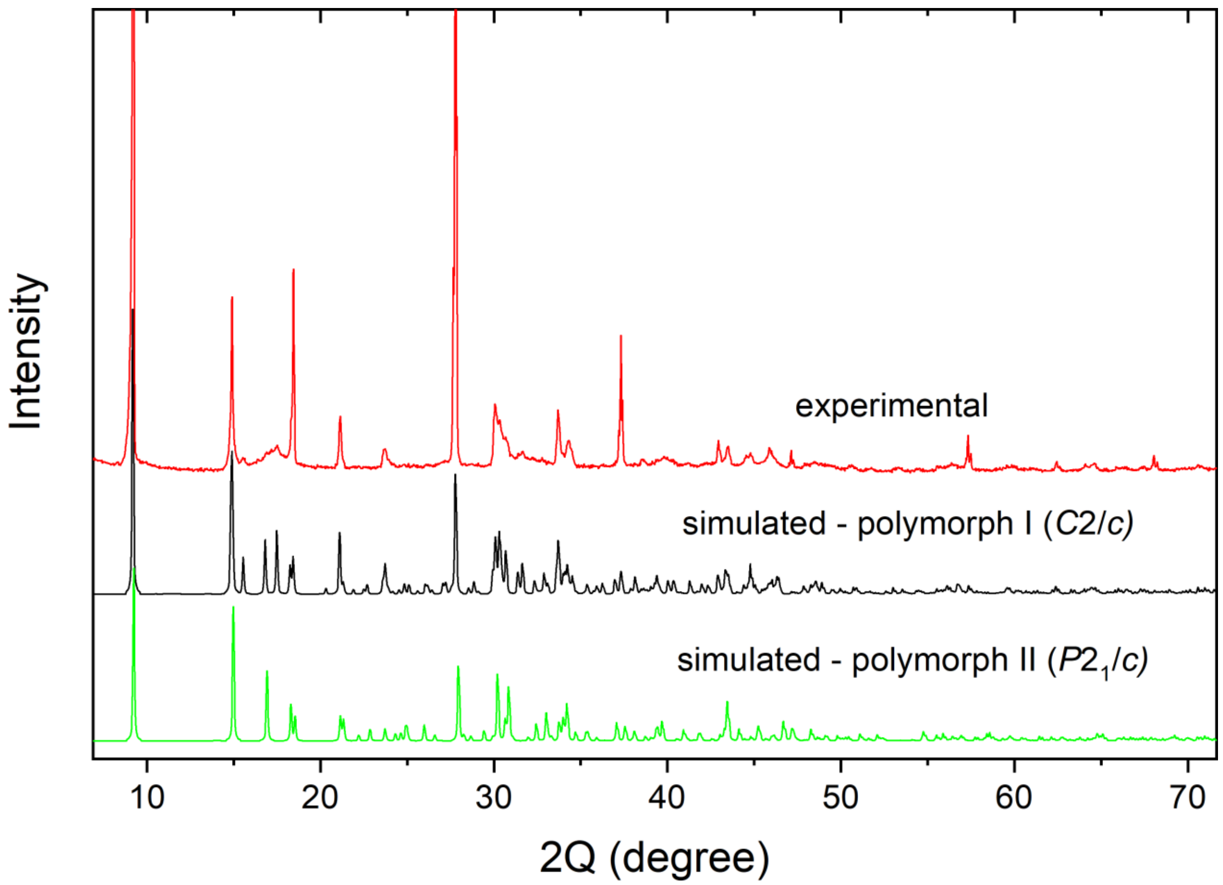

Figure S1. Experimental and simulated powder X-ray diffraction patterns of Tz_2PbBr_4.

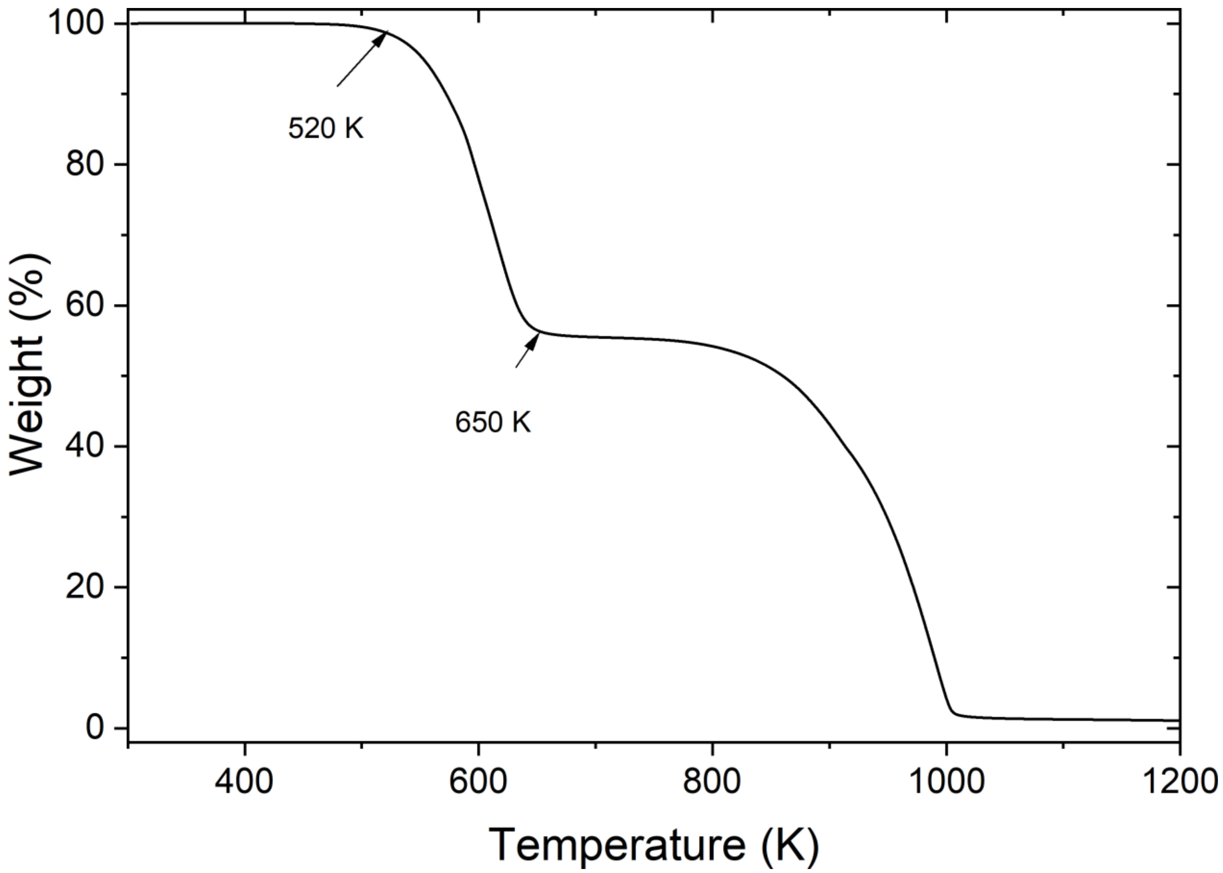

Figure S2. TG plot of Tz_2PbBr_4.

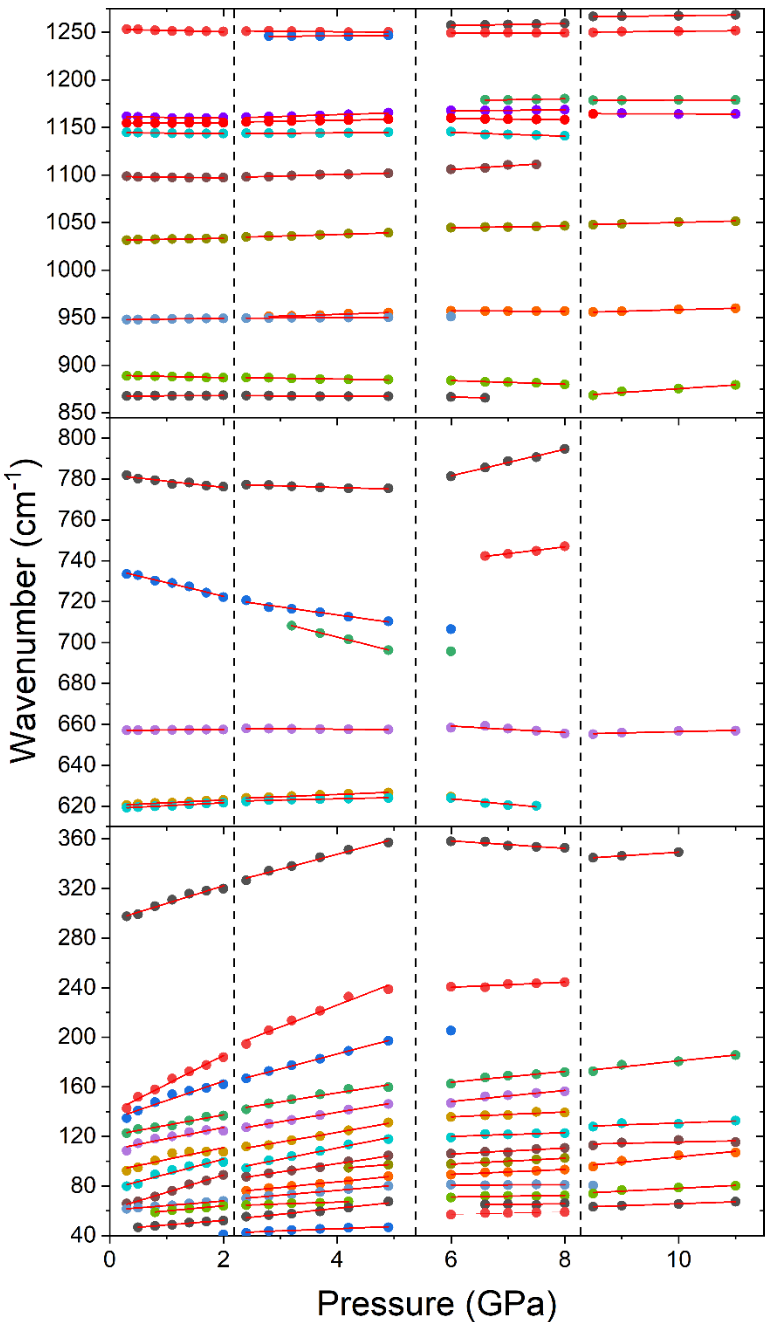

Figure S3. Pressure dependence of the Raman modes during the compression run. Solid lines are linear fits on the data to $\omega(P) = \omega_0 + \alpha P$. Vertical lines correspond to the phase transition pressure.

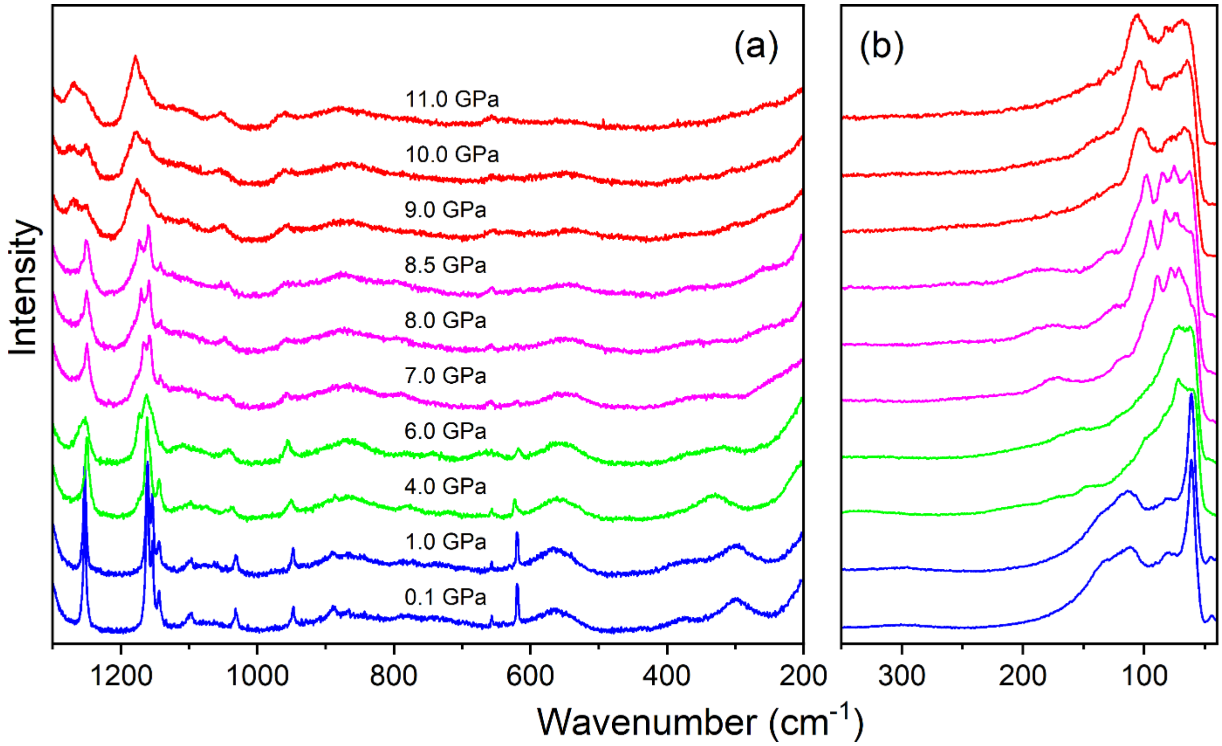

Figure S4. Raman spectra of Tz_2PbBr_4 during decompression run. Blue, green, magenta and red colors denote ambient pressure phase I and HP phases II, III and IV, respectively.

Table S1. Wavenumber intercepts at zero pressure (ω_0) and pressure coefficients ($\alpha = d\omega/dP$), obtained from fitting of the experimental data by linear functions, for the four phases of Tz_2PbBr_4.

ambient pressure phase I		HP phase II		HP phase III		HP phase IV		Assignment*
ω_0 (cm^{-1})	α ($cm^{-1}GPa^{-1}$)	ω_0 (cm^{-1})	α ($cm^{-1}GPa^{-1}$)	ω_0 (cm^{-1})	α ($cm^{-1}GPa^{-1}$)	ω_0 (cm^{-1})	α ($cm^{-1}GPa^{-1}$)	
				1251.8	0.91	1260.5	0.71	δC-H
1253.5	-1.49	1252.6	-0.50	1250.1	-0.08	1245.0	0.62	δC-H
		1245.1	0.29					δC-H
				1172.1	1.00	1177.6	0.12	νC-N, δC-H
1161.4	-0.77	1156.1	1.84	1164.9	0.45	1165.8	-0.16	νC-N, δC-H
1154.4	0.32	1153.2	1.08	1164.5	-0.81			δC-H, νC-N
1144.8	-0.70	1142.7	0.40	1156.6	-1.96			δC-H, νC-N
1098.6	-0.80	1094.2	1.59	1082.9	3.83			νN-N
1031.5	0.97	1030.6	1.77	1039.3	0.88	1035.6	1.46	δNNC, δC-H
		945.8	1.94	957.3	-0.05	942.2	1.62	δNCN
947.7	0.82	948.7	0.33					δNCN
889.4	-1.25	889.4	-0.97	895.3	-1.91	835.0	4.03	δCNN, δNNC
867.6	0.26	868.9	-0.35	876.3	1.60			γC-H ip
782.0	-3.06	779.1	-0.81	742.5	6.51			γN-H ip
				720.3	3.32			γN-H icp
736.0	-6.65	729.1	-3.88					γN-H icp
		730.5	-6.96					γN-H icp
657.0	0.23	658.4	-0.19	668.4	-1.54	649.9	0.65	γ_{ring} CNNC
620.3	1.36	621.3	1.10					γ_{ring} CNCN
618.8	1.45	621.1	0.62	639.0	-2.58			γ_{ring} CNCN
293.8	14.22	299.4	12.08	376.5	-3.00	319.7	2.96	T'(Tz^+)

138.7	23.33	155.1	17.74	227.7	2.11			Pb-Br stretch+ L+ T’
133.3	15.68	138.6	11.98					Pb-Br stretch+ L+ T’
120.7	8.38	125.9	7.31	137.7	4.35	132.9	4.81	Pb-Br stretch+ L+ T’
109.1	9.09	108.9	7.65	121.1	4.51			Pb-Br stretch+ L+ T’
91.7	9.65	91.4	8.00	123.8	1.99			Pb-Br stretch+ L+ T’
77.6	12.12	74.1	9.16	110.1	1.62	115.0	1.59	Pb-Br bend
61.0	13.90	70.9	6.80	91.4	2.41	105.5	0.99	Pb-Br bend
		80.4	3.43	83.1	2.43			Pb-Br bend
		65.3	4.54	77.3	2.01	59.4	4.42	Pb-Br bend
60.5	3.68	60.6	4.00	80.2	0.11			Pb-Br bend
55.7	4.17	60.9	1.55	67.1	0.67	54.5	2.37	Pb-Br bend
44.8	3.73	42.8	4.82	61.7	0.50	49.5	1.62	Pb-Br bend
		38.4	1.79	51.5	0.95			L($PbBr_6$)

*ν, δ, γ, T’ and L denote stretching, in-plane bending, out-of-plane bending, translational and librational modes of Tz^+.

Vibrational modes should be assigned to internal modes of Tz^+ cation, lattice modes of Tz^+ cation (translational and librational modes) and vibrations of the inorganic substructure.

Internal modes: for an isolated Tz^+ cation, 21 modes are expected (M. Mączka, S. Sobczak, M. Ptak, S. Smółka, K. Fedoruk, F. Dybała, A. P. Herman, W. Paraguassu, J. K. Zaręba, R Kudrawiec, A. Sieradzki and A. Katrusiak, Revisiting a (001)-Oriented Layered Lead Chloride Templated by 1,2,4-Triazolium: Structural Phase Transitions, Lattice Dynamics and Broadband Photoluminescence, *Dalton Trans.* 2024, **53**, 6906−6919; M. Daszkiewicz, Crystal Structure, Vibrational and Theoretical Studies of bis(1,4-H_2-1,2,4-triazolium)hexachloridostanate(IV) monohydrate, *J. Mol. Struct*., 2013, **1032**, 56-61). However, only 13 of them should appear in the 1300-600 cm^{-1} range. Our Raman study shows 14 bands at ambient conditions because the single γ_{ring} CNCN mode of an isolated Tz^+ cation splits into two components at 618.8+620.3 cm^{-1} due to presence of two independent Tz^+ cations in polymorph I. The two expected γN-H modes are easily identified at 736 and 782 cm^{-1} since the corresponding Raman bands are much broader than all remaining internal bands. The remaining internal modes have been assigned by comparison with the chloride analogues because their Raman spectra are qualitatively very similar in the 1300-850 cm^{-1} range, except of shift of the Raman bands to lower wavenumbers by 3-10 cm^{-1} when Cl is replaced by Br.

Vibrations of the inorganic substructure and lattice modes of Tz^+. Raman studies of layered lead bromides showed that the lowest wavenumber modes (below 40 cm^{-1}) should be assigned to L($PbBr_6$) (called often octahedron rocking/twisting, see M. Mączka and M. Ptak, Lattice Dynamics and Structural Phase Transitions in Two-Dimensional Ferroelectric Methylhydrazinium Lead Bromide Investigated Using Raman and IR Spectroscopy*, J. Phys. Chem. C,* 2022, **126**, 7991−7998; R. Krahne, M. L. Lin and P. H. Tan, Interplay of Phonon Directionality and Emission Polarization in Two-Dimensional Layered Metal Halide Perovskites, *Acc. Chem. Res.,* 2024, **57**, 2476−2489). At ambient conditions, this mode is located below our experimental limit of 40 cm^{-1} but it appears at higher pressure with ω_0 38.4 cm^{-1}. Pb-Br bending

modes are reported in literature as the most intense Raman bands in the 40-85 cm^{-1} range. Therefore, we attribute all bands of Tz_2PbBr_4 in the 40-80 cm^{-1} (ambient pressure phase) to these modes. Note that on compression these modes exhibit significant shift to higher wavenumbers. Literature data showed that the third region, 80-140 cm^{-1}, is dominated by Pb-Br stretching modes. Raman bands in this region are usually much weaker than those corresponding the Pb-Br bending modes. We attribute to these modes all Raman bands observed for the ambient pressure phase in the 90-140 cm^{-1} range. Note that these modes exhibit very strong pressure dependence. Finally, one would expect to observe lattice modes of Tz^+ cation. We attribute the band near 294 cm^{-1} to T'(Tz^+). It is worth noting that intensity of this band is an order of magnitude smaller compared to the Pb-Br stretching modes (see Figure 2c). Therefore, the remaining Raman bands corresponding to the lattice modes are overlapped by much stronger bands arising from the Pb-Br stretching modes. We have indicated this in Table S1 by putting T' and L symbols after Pb-Br stretch.
